# Preliminary evaluation of a fractal-based collateral biomarker for thrombectomy candidate selection in acute ischemic stroke: a retrospective study

**DOI:** 10.7717/peerj.21045

**Published:** 2026-04-13

**Authors:** Chien-Hung Chang, Wen-Piao Lin, Ching-Chang Chen, Kuo-Hsuan Chang

**Affiliations:** 1 Department and Graduate Institute of Electrical Engineering, College of Engineering, Chang Gung University, Taoyuan, Taiwan; 2 Department of Neurology, Chang Gung University College of Medicine, Chang Gung Memorial Hospital, Linkou Branch, Chang Gung University, Taoyuan, Taiwan; 3 Department of Neurosurgery, Linkou Medical Center, Chang Gung Memorial Hospital, Chang Gung University, Taoyuan, Taiwan

**Keywords:** Acute ischemic stroke, Collateral circulation, Fractal dimension, Computed tomography angiography, Endovascular thrombectomy

## Abstract

**Background:**

Collateral circulation plays a supportive role in stroke recovery, particularly among patients undergoing endovascular thrombectomy (EVT). While multiphase computed tomography angiography (mCTA)-based visual collateral scoring (vCS-mCTA) is widely used, its subjectivity and interobserver variability limit consistency. This preliminary study aimed to evaluate whether the mean fractal dimension ratio (mFD), derived from single-phase CTA (sCTA)-equivalent images, could serve as an objective and reproducible biomarker for collateral status assessment.

**Materials and Methods:**

This study retrospectively analyzed 54 patients with acute ischemic stroke who underwent EVT. The mFD was calculated using semi-automated fractal analysis of segmented vessels from the first phase of mCTA images. Collateral status was assessed using both sCTA and mCTA Menon visual scoring systems. The primary outcome was favorable 90-day functional status (modified Rankin Scale ≤ 2).

**Results:**

The sCTA-based visual collateral score (vCS-sCTA) was not significantly associated with clinical outcome. In contrast, higher mFD were significantly associated with favorable outcomes and demonstrated concordance with vCS-mCTA. In multivariable analysis, mFD remained an independent predictor after adjusting for clinical covariates. Its predictive performance was comparable to vCS-mCTA and superior to single-phase visual scoring.

**Conclusion:**

The mFD derived from sCTA-equivalent images may offer a practical and reproducible imaging biomarker for collateral assessment, offering predictive performance comparable to vCS-mCTA. As it does not rely on multiphase acquisition or subjective interpretation, mFD may enhance thrombectomy candidate selection, particularly in resource-limited or time-constrained settings.

## Introduction

Acute ischemic stroke (AIS) remains a leading cause of disability and mortality worldwide (Virani et al., 2020), with cerebral artery occlusion or embolism from the heart or neck vessels being primary etiologies (Adams Jr et al., 1993). Pial collaterals play a critical role in sustaining tissue perfusion and reducing ischemic injury (Liebeskind, 2005). Experimental studies (Zhang et al., 2010) have shown that features such as collateral density, vessel diameter, and arteriole count correlate more closely with infarct volume than overall cerebral artery territory. These findings underscore that the morphological characteristics of collateral networks—such as density and branching complexity—may better predict infarct progression and neurological outcomes than the anatomical extent of occlusion. While clinical and imaging factors like infarct size and location are central to determining eligibility for endovascular thrombectomy (EVT), timely and precise collateral assessment offers complementary prognostic value (Chen et al., 2024) and can enhance treatment decision-making.

Multiphase computed tomography angiography (mCTA) (He et al., 2021) is widely used to assess collateral status, with the mCTA-derived visual collateral score (vCS-mCTA) providing a qualitative evaluation based on clinician interpretation. Despite mCTA improves temporal resolution, single-phase CTA (sCTA) remains the predominant imaging modality in many clinical settings. The sCTA-derived visual collateral score (vCS-sCTA), while informative, is limited by its inability to detect delayed collateral filling (Lu et al., 2022; Boers et al., 2018). Furthermore, both vCS-mCTA and vCS-sCTA are inherently subjective, leading to considerable inter-observer variability and reduced reproducibility. These limitations highlight the need for an objective, standardized alternative—such as a quantitative collateral score (qCS)—to support clinical decision-making in a consistent manner.

Fractal Dimension (FD), which quantifies morphological complexity, has been extensively applied in medical imaging to characterize microvascular networks (Aliahmad et al., 2014; Doubal et al., 2010; Khan et al., 2022). In neurovascular research, FD analysis has primarily focused on retinal vasculature as a stroke biomarker, where lower FD values correlate with sparse branching and a higher risk of cerebrovascular disease (Khan et al., 2022). However, its applications in cerebral arteries remain limited, primarily explored in the context of arteriovenous malformation (AVM) classification (Reishofer et al., 2012). FD analysis in AVM has shown that collateral vessel morphology shares hierarchical branching features with retinal networks, aligning with the principles of fractal geometry. This finding highlights its potential as a quantitative tool for assessing brain collateral circulation.

To overcome the subjectivity and variability of visual collateral scoring (vCS), this preliminary study explored a semi-automated method to derive the mean fractal dimension ratio (mFD) from the first phase of mCTA, corresponding to sCTA-equivalent data commonly available in clinical settings. This study aimed to evaluate whether mFD could serve as a reproducible imaging biomarker for collateral assessment and enhance clinical decision-making, particularly in settings where mCTA is unavailable or incomplete.

## Materials & Methods

This study retrospectively analyzed AIS patients with anterior large vessel occlusion (LVO) who presented within 8 h of onset and underwent EVT at Chang Gung Memorial Hospital between April 2015 and Feb 2016. All patient-identifying information was anonymized in accordance with the ethical principles and institutional guidelines. The study was approved by the Chang Gung Medical Foundation Institutional Review Board (IRB No. 202201661BD0001). Written informed consent was waived due to the retrospective nature of the study and the exclusive use of de-identified imaging data for analysis. The study period (April 2015–February 2016) was deliberately chosen to capture a transitional phase in our institutional imaging workflow. During this period, single-phase CTA (sCTA) was initially the standard modality for acute ischemic stroke evaluation, and multiphase CTA (mCTA) was gradually introduced to the clinical protocol. This timeframe allowed for consistent acquisition and comparable image quality across patients, while still reflecting the evolving adoption of mCTA in clinical practice. The use of this dataset enabled a methodologically coherent comparison of quantitative and visual collateral assessments based on equivalent first-phase arterial imaging. Inclusion criteria were patients older than 18 years with a premorbid modified Rankin Scale (mRS) ≤ 2 and occlusion involving the M1 segment of the middle cerebral artery (MCA) and/or the intracranial internal carotid artery (ICA). Exclusion criteria were significant contralateral vascular stenosis (≥70% luminal stenosis or complete occlusion of the contralateral ICA or proximal MCA on CTA) that could compromise hemodynamic or collateral assessment, intracranial hemorrhage (ICH) on non-contrast CT, an Alberta Stroke Programme Early CT Score (ASPECTS) <6, prior infarction involving more than one-third of the contralateral MCA territory, CTA images with motion artifacts or improper phase, Moyamoya disease, or incomplete clinical/imaging data. CTA examinations were performed as part of a combined head-and-neck protocol using bolus tracking, with the region of interest (ROI) placed in the proximal descending aorta and a trigger threshold >180 Hounsfield units (HU) to ensure consistent arterial-phase timing. For both protocols, sCTA and the first phase of mCTA were acquired using this same bolus-tracking technique. The FD analysis was performed on these first-phase arterial images derived from mCTA acquisitions, which were considered equivalent to sCTA based on comparable acquisition timing. However, we acknowledge that these images may not be fully equivalent with routine sCTA acquired under different protocols. Of the 65 patients initially screened, 54 were included after excluding those with prior large strokes, poor imaging quality, or incomplete imaging data (*e.g.*, missing later phases due to motion or agitation). Clinical risk factors, laboratory data, and advanced brain imaging were collected. Functional outcome at 90 days was measured using the modified Rankin Scale (mRS), with favorable outcome defined as mRS ≤ 2. Because large-vessel occlusion (LVO) is typically attributable to either cardioembolism or atherosclerotic disease, small-vessel disease was not present in this cohort. In the acute reperfusion workflow, rapid treatment is prioritized, and detailed etiologic differentiation between extracranial LAA and intracranial atherosclerotic disease (ICAD) is not routinely performed. Therefore, cardioembolic (CE) stroke was recorded when identifiable, while all remaining non-CE LVOs were categorized as atherosclerotic mechanisms.

vCS was independently performed by two board-certified physicians (a neuroradiologist and a neurologist), each with over 10 years of experience, using both the single-phase and multiphase Menon scoring systems (Menon et al., 2015). The raters (Dr. Wu and Dr. Lin) were blinded to all clinical data. Both methods grade collateral status on a 6-point scale from 0 (no visible collaterals) to 5 (normal or increased pial vessels compared to the contralateral hemisphere). The single-phase score assesses vessel extent at a single time point, whereas the multiphase score incorporates temporal dynamics across three CTA phases, providing a more comprehensive evaluation of collateral recruitment. For multiphase CTA, all three phases were jointly reviewed, and a single consensus score was determined to represent the best overall collateral extent, rather than averaging the scores across phases. This approach reflects real-world clinical practice, in which the phase showing the most robust arterial filling is prioritized for interpretation. Scoring discrepancies were resolved through consensus discussion between the two raters. To facilitate comparison and subgroup analysis, vCS-sCTA and vCS-mCTA were categorized into predefined strata based on previously established thresholds (Menon et al., 2015). Specifically, scores of 0 to 1 were classified as severe, 2 to 3 as moderate, and 4 to 5 as good. This stratification was intended to reflect clinically meaningful differences in collateral status and to simplify cross-modality comparison.

Collateral data processing was conducted using a Python-based pipeline that integrated FracLac source code from ImageJ and consisted of three main steps: (1) Preprocessing: First-phase CTA, extracted from the mCTA acquisition, was preprocessed using adaptive thresholding to enhance vessel contrast and suppress noise. (2) FD Analysis: Box-counting FD values were semi-automatically computed for both the symptomatic and contralateral hemispheres at two anatomical levels: ganglionic and supraganglionic. To ensure reproducibility, two axial sections—ganglionic and supraganglionic levels—were selected for FD computation based on optimal arterial-phase contrast and minimal venous contamination. Because the presence of early venous opacification can artificially increase vascular complexity and thus elevate FD values, slices were chosen where arterial delineation was clear and venous filling was minimal. This semi-automated selection ensured consistent segmentation quality and minimized intersubject variability across cases. The analysis encompassed the proximal M1 to M2 branches within the middle cerebral artery (MCA) territory. FD ratios were calculated by dividing the FD of the symptomatic hemisphere by that of the contralateral side for each level. (3) Mean FD Ratio (mFD): The mFD was defined as the average of the two FD ratios, reflecting both spatial complexity and hemispheric symmetry. mFD were stratified into quartiles (Q1–Q4) based on their distribution within the study cohort, with Q4 indicating the highest and Q1 the lowest degree of vascular complexity. This stratification was used to assess functional outcome distribution and to examine concordance between mFD and vCS.

All statistical analyses were conducted using Python (Version 3.10) with open-source packages. Continuous variables were analyzed with the Student’s *t*-test or Mann–Whitney U test as appropriate; categorical data were evaluated using the chi-square test or Fisher’s exact test. For within-subject comparisons (*e.g.*, vCS-sCTA *vs.* vCS-mCTA), the Wilcoxon signed-rank test was applied. Spearman’s rank correlation was used to assess the association between mFD and vCS-mCTA. Receiver operating characteristic (ROC) analysis was conducted to determine the optimal mFD cutoff for predicting favorable outcomes. Using Youden’s index, the optimal threshold for mFD was identified as >0.9307, yielding a sensitivity of 73.9% and a specificity of 71.0%. The discriminative abilities of mFD and vCS-mCTA were compared using DeLong’s test for paired ROC curves. Variables significant in univariate analysis were entered into a multivariate logistic regression model, adjusting for age, gender, pre-thrombectomy NIH Stroke Scale score (pre-NIHSS), and low-density lipoprotein (LDL) levels. No correction for multiple comparisons was applied due to the exploratory nature of this preliminary analysis; *P* < 0.05 was considered statistically significant.

## Results

A total of 54 AIS patients were included in the study (mean age: 65.13 ± 14.16 years; 64.8% male). As shown in Table 1, patients with favorable outcomes (mRS ≤ 2) had lower pre-NIHSS scores (*P* = 0.002) and lower LDL levels (*P* = 0.033). There was a trend toward lower prevalence of dyslipidemia (*P* = 0.085) among patients with favorable outcomes. No statistically significant differences were observed in in age (*P* = 0.260), gender (*P* = 0.135), hypertension (*P* = 0.386), the rates of EVT (*P* = 0.517) or bridging therapy with IVT followed by EVT (*P* = 0.517) between patients with favorable and unfavorable outcomes. Post-EVT intracranial hemorrhage (ICH) occurred in 7 of 54 patients (13.0%), including 2 cases (8.3%) in the favorable outcome group and 5 cases (16.7%) in the unfavorable outcome group. The difference between groups was not statistically significant (*P* = 0.43).

**Table 1:** Characteristics of acute ischemic stroke patients undergoing thrombectomy: comparison between favorable and unfavorable outcomes.

**Variable** **AIS risk factor**	**All, *N* = 54**	**Favorable (mRS**≤**2), *N* = 24**	**Unfavorable (mRS** >**2), N=30**	***P*-value**
Age, years	65.13 ± 14.16	62.61 ± 13.59	67.00 ± 14.51	0.260
Gender, Male, n (%)	35 (64.8%)	18 (78.3%)	17 (54.8%)	0.135
Dyslipidemia, n (%)	9 (16.7%)	1 (4.3%)	8 (25.8%)	0.085
Hypertension, n (%)	26 (48.1%)	9 (39.1%)	17 (54.8%)	0.386
Pre-NIHSS	18.30 ± 6.93	15.00 ± 6.23	20.74 ± 6.47	0.002^**^
Cholesterol (mg/dL)	173.85 ± 40.57	167.35 ± 31.58	178.68 ± 46.05	0.289
HbA1C (%)	6.66 ± 1.49	6.43 ± 1.04	6.83 ± 1.75	0.307
LDL (mg/dL)	111.78 ± 37.65	99.57 ± 31.75	120.84 ± 39.56	0.033^*^
Creatinine (mg/dL)	1.02 ± 0.397	0.95 ± 0.192	1.08 ± 0.495	0.185
Post-EVT hemorrhage, n(%)	(13.0%)	(8.3%)	(16.7%)	0.43
**Thrombectomy parameters**				
EVT, n (%)	34 (60.7%)	14 (56.0%)	20 (64.5%)	0.517
EVT+IVT, n (%)	22 (39.3%)	11 (44.0%)	11 (35.5%)	0.517
TICI score				0.682
0-2a, n (%)	6 (11.1%)	2 (8.3%)	4 (13.3%)	
≥2b, n (%)	48 (88.9%)	22 (91.7%)	26 (86.7%)	
Onset-to-groin puncture time (min) medians, IQR	285 (169–435)	267 (153–414)	307 (181–455)	0.765
Procedure duration (min) medians, IQR	55 (40–75)	50 (39–64)	60 (43–88)	0.497
Number of passes, medians, IQR	2 (1–3)	2 (1–2)	2 (1–3)	0.328
**Imaging biomarkers**				
vCS-sCTA	3.11 ± 0.84	3.35 ± 0.78	2.94 ± 0.85	0.074
vCS-mCTA	2.89 ± 0.93	3.30 ± 0.77	2.58 ± 0.92	0.003^**^
ASPECTs	8.54 ± 1.59	8.52 ± 1.73	8.55 ± 1.50	0.952
mFD ratio	0.89 ± 0.11	0.93 ± 0.09	0.86 ± 0.12	0.017^*^

**Notes.**

* *p* < 0.05.

** *p* < 0.01.

Abbreviations EVT endovascular thrombectomy IVT Intravenous Thrombolysis TICI Thrombolysis in Cerebral Infarction Pre-NIHSS Pre-EVT National Institutes of Health Stroke Scale LDL Low-Density Lipoprotein HbA1C Glycated Hemoglobin vCS-sCTA sCTA-derived visual collateral score vCS-mCTA mCTA-derived visual collateral score mFD mean fractal dimension ratio ASPECTs Alberta Stroke Program Early CT Score IQR interquartile range

Collateral status was assessed using the mFD and vCS-mCTA, revealed significant differences between outcome groups. Patients with favorable outcomes had a higher mFD (0.931 ± 0.086 *vs.* 0.863 ± 0.118, *P* = 0.017) and higher vCS-mCTA scores (3.30 ± 0.77 *vs.* 2.58 ± 0.92, *P* = 0.003). A Wilcoxon signed-rank test confirmed that vCS-mCTA scores were significantly higher than vCS-sCTA (*Z* = −2.191, *P* = 0.028), and Spearman correlation showed a moderate positive association between mFD and vCS-mCTA (*ρ* = 0.580, *P* < .001), indicating concordance between quantitative and visual assessments.

**Figure 1:**
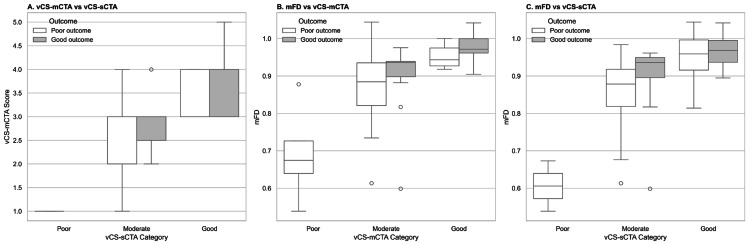
Relationship between visual collateral scores, mFD, and clinical outcomes. (A) Comparison between vCS-sCTA and vCS-mCTA. Patients classified as moderate on vCS-sCTA showed wide variability in vCS-mCTA scores, suggesting that sCTA may underestimate collateral extent. (B) Association between mFD and vCS-mCTA. mFD values increased progressively across higher vCS-mCTA categories, demonstrating strong concordance with multiphase collateral grading. (C) Association between mFD and vCS-sCTA. mFD enabled further prognostic stratification within vCS-sCTA categories—particularly among moderate cases—revealing favorable perfusion patterns not captured by visual assessment alone. Abbreviations: vCS-sCTA, visual collateral score based on single-phase CTA; vCS-mCTA, visual collateral score based on multiphase CTA; mFD, mean fractal dimension ratio.

To assess the distribution patterns of collateral scoring methods in relation to clinical outcomes, Fig. 1 presents boxplots illustrating their distributions across predefined vCS categories and 90-day outcomes (mRS ≤ 2). In Panel A, patients grouped as “Moderate” by vCS-sCTA showed wide dispersion in their vCS-mCTA scores, with outcome overlap across the entire range. Panel B demonstrates a near-linear relationship between vCS-mCTA categories and mFD values, with patients achieving favorable outcomes tending to exhibit higher mFD values within each category. Panel C reveals a noteworthy trend. Among patients classified within the same vCS-sCTA category, those in the moderate group with higher mFD values had a greater likelihood of favorable outcomes.

In univariate logistic regression (Table 2), both mFD > 0.9307 (OR = 6.93, 95% CI [2.06–23.26], *P* = 0.002) and vCS-mCTA (OR = 2.77, 95% CI [1.31–5.85], *P* = 0.008) were significantly associated with favorable outcomes. After adjusting for age, gender, LDL level, and pre-NIHSS score, both remained independent predictors: mFD > 0.9307 (adjusted OR = 8.13, 95% CI [1.86–35.54], *P* = 0.005) and vCS-mCTA (adjusted OR = 3.32, 95% CI [1.31–8.36], *P* = 0.011). Receiver Operating Characteristic (ROC) analysis showed that mFD had an AUC of 0.72 (95% CI [0.63–0.80], *P* < 0.001), comparable to vCS-mCTA (AUC = 0.71, 95% CI [0.58–0.83], *P* = 0.01). DeLong’s test for paired ROC curves indicated no statistically significant difference between the two methods (*P* = 0.847). In contrast, vCS-sCTA was not significantly associated with favorable 90-day outcomes (*P* = 0.074).

**Table 2:** Comparative multivariable logistic regression models and diagnostic performance for predicting favorable outcomes (mRS ≤ 2).

**Variable**	**OR (95% CI)**	***P***	**OR (95% CI)**	***P***
	**mFD** > **0.9307**		**vCS-mCTA**	
**Crude** ** (unadjusted)**				
mFD > 0.9307	6.93 (2.06–23.26)	0.002^**^	–	-
vCS-mCTAs	–	–	2.77 (1.31–5.85)	0.008^**^
AUC (95% CI)	0.72 (0.63–0.80)	<0.001^**^	0.71 (0.58–0.83)	0.010^*^
Sensitivity, %	73.9%		83.9%	
**Adjusted for covariates**	**Model 1 OR (95% CI)**		**Model 2 OR (95% CI)**	
mFD *>* 0.9307	8.13 (1.86–35.54)	0.005^**^	–	-
vCS-mCTA	–	–	3.32 (1.31–8.36)	0.011^*^
Age	0.99 (0.94–1.04)	0.60	0.98 (0.93–1.04)	0.532
Gender (Male)	3.82 (0.78–18.73)	0.10	5.17 (1.01–26.43)	0.048^*^
Pre-NIHSS	0.86 (0.74–0.99)	0.03^*^	0.87 (0.76–1.00)	0.05
LDL	0.98 (0.97–1.00)	0.12	0.98 (0.96–1.00)	0.125
AUC (95% CI)	0.87 (0.77–0.96)	<0.001^**^	0.85 (0.74–0.94)	<0.001^**^
Hosmer–Lemeshow Test (Chi-square)	Chi-sq = 12.78, *df* = 8, *P* = 0.120		Chi-sq = 6.47, *df* = 8, *P* = 0.595	
Akaike Information Criterion (AIC)	60.48 (Model 1)		61.22 (Model 2)	

**Notes.**

* *P* < 0.05.

** *P* < 0.01.

Model definitions: Model 1 includes mFD ¿0.9307 as the primary predictor; Model 2 includes vCS-mCTA . Both models were adjusted for age, gender, baseline NIHSS, and LDL levels. Performance metrics: AUC and sensitivity are reported to facilitate comparison of diagnostic discrimination between models. Statistical tests: Hosmer–Lemeshow test and Akaike Information Criterion (AIC) were used to assess goodness of fit.

Abbreviations mFD mean fractal dimension ratio vCS-mCTA visual collateral score based on multiphase CTA Pre-NIHSS baseline National Institutes of Health Stroke Scale score LDL low-density lipoprotein OR odds ratio CI confidence interval AUC area under the receiver operating characteristic curve

## Discussion

This preliminary study explores a quantitative, fractal-based approach to collateral assessment as a potential complement to existing stroke imaging methods. Specifically, this study assessed whether the mFD, calculated from sCTA-equivalent images, could serve as a reliable and objective imaging marker to assist in EVT candidate selection. Compared with conventional vCS-mCTA, which is subjective to inter-observer variability, mFD provides a standardized quantitative measure. Our findings demonstrate that mFD is not only associated with favorable outcomes (mRS ≤ 2), but also remains an independent predictor after adjustment for age, sex, LDL, and pre-treatment NIHSS. Notably, despite being derived from sCTA, mFD demonstrated predictive performance comparable to vCS-mCTA, suggesting its potential applicability in settings where mCTA is unavailable.

Analysis of clinical and procedural characteristics (Table 1) reveals that lower pre-NIHSS scores and lower LDL levels are significantly associated with favorable outcomes. In contrast, age and gender did not reach statistical significance. Procedural metrics, such as onset-to-groin puncture time and thrombectomy duration, were not significantly associated with clinical outcomes. Notably, both vCS-mCTA (*P* = 0.003) and mFD (*P* = 0.017) showed significant associations with favorable outcomes. This finding aligns with previous studies (Bang et al., 2011; Anadani et al., 2021), emphasizing the supportive role of collateral status in achieving successful reperfusion and improved functional recovery. In contrast, vCS-sCTA (*P* = 0.074) did not reach statistical significance in predicting outcomes, likely due to its limited ability to capture delayed collateral recruitment with single-phase imaging. Prior studies (Boers et al., 2018) have shown that sCTA tends to underestimate collateral capacity, which may result in misclassification of patients who could benefit from EVT. However, the non-significant result could also be attributed, at least in part, to limited statistical power arising from our modest sample size. This limitation has been acknowledged as a constraint of the current study.

Importantly, both vCS-sCTA and mFD were derived from the same sCTA-equivalent phase of mCTA. Nevertheless, mFD demonstrated significantly predictive value, approaching the performance of vCS-mCTA. Figure 1 further illustrates these differences. In Panel A, patients classified as “Moderate” or “Good” by vCS-sCTA exhibited wide variability in both vCS-mCTA scores and clinical outcomes, suggesting limited prognostic accuracy of static-phase visual assessment. For instance, a patient with a vCS-sCTA score of 2 may appear ineligible for EVT, whereas mCTA could reveal a vCS-mCTA score of 4, reflecting delayed yet adequate collateral filling not captured on static imaging. Without temporal resolution, such patients risk misclassification and potential exclusion from beneficial intervention. In contrast, Panel B demonstrates a clear upward trend in mFD values across increasing vCS-mCTA categories, with favorable outcomes predominantly clustering at higher mFD levels. This correlation highlights the close correspondence between mFD and time-resolved mCTA assessment, reinforcing mFD’s potential as a surrogate imaging marker. Panel C reveals a clinically meaningful trend. Among patients with the same vCS-sCTA classification, especially those in the moderate group, mFD allows for further stratification of outcomes. Patients with higher mFD values, particularly those in the upper quartiles (Q3–Q4), were more likely to achieve favorable outcomes despite having ambiguous vCS. This finding suggests that mFD may capture subtle spatial or geometric details, such as branching complexity or mild contrast distribution irregularities, which are often overlooked in static vCS. In this context, mFD may serve as a valuable adjunct for refining prognostic evaluation in borderline cases where vCS alone is inconclusive. mFD provides an observer-independent and quantitative measure derived from static CTA. It bridges the gap between vCS-sCTA and vCS-mCTA, enabling more consistent collateral evaluation when mCTA is not feasible. This is especially relevant for patients with contraindications to contrast or agitation during imaging. Although derived from the same sCTA-equivalent images, mFD extracts additional structural information that improves both collateral classification and outcome prediction.

**Figure 2:**
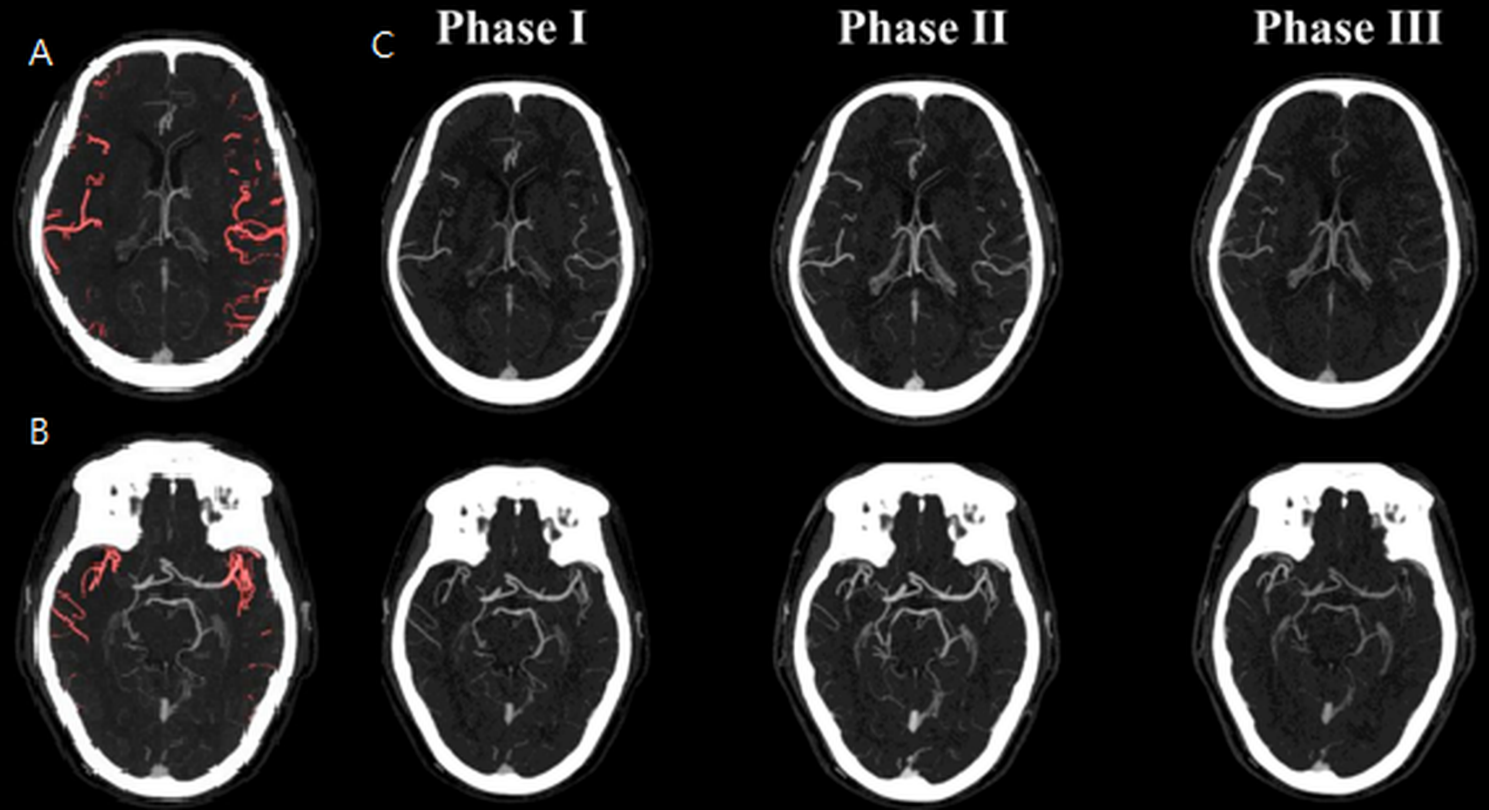
Representative axial CTA slices illustrating the fractal dimension (FD) analysis workflow in comparison to conventional visual collateral scoring (vCS). (A, B) Phase I CTA images at the ganglionic and supraganglionic levels, respectively, showing the semi-automated vessel segmentation applied for FD computation. (C) Multiphase CTA images (Phases I–III) at corresponding levels, used for both vCS-sCTA and vCS-mCTA assessments. FD analysis was performed exclusively on Phase I images—serving as an sCTA-equivalent image—with a focus on the bilateral MCA M1–M2 segments. The mean FD ratio (mFD) was calculated as the average of the symptomatic-to-contralateral FD ratios across both anatomical levels.This case involves a 57-year-old male with right MCA occlusion (NIHSS = 19) who achieved successful reperfusion (TICI III) and a favorable outcome (90-day mRS = 0). Notably, the collateral assessments were discordant: vCS-sCTA = 2, vCS-mCTA = 4, and mFD = 0.871. Abbreviations: vCS-sCTA, visual collateral score based on single-phase CTA; vCS-mCTA, visual collateral score based on multiphase CTA; FD, fractal dimension; mFD: mean fractal dimension ratio; MCA, middle cerebral artery; NIHSS, National Institutes of Health Stroke Scale; TICI, Thrombolysis in Cerebral Infarction; mRS, modified Rankin Scale.

**Figure 3:**
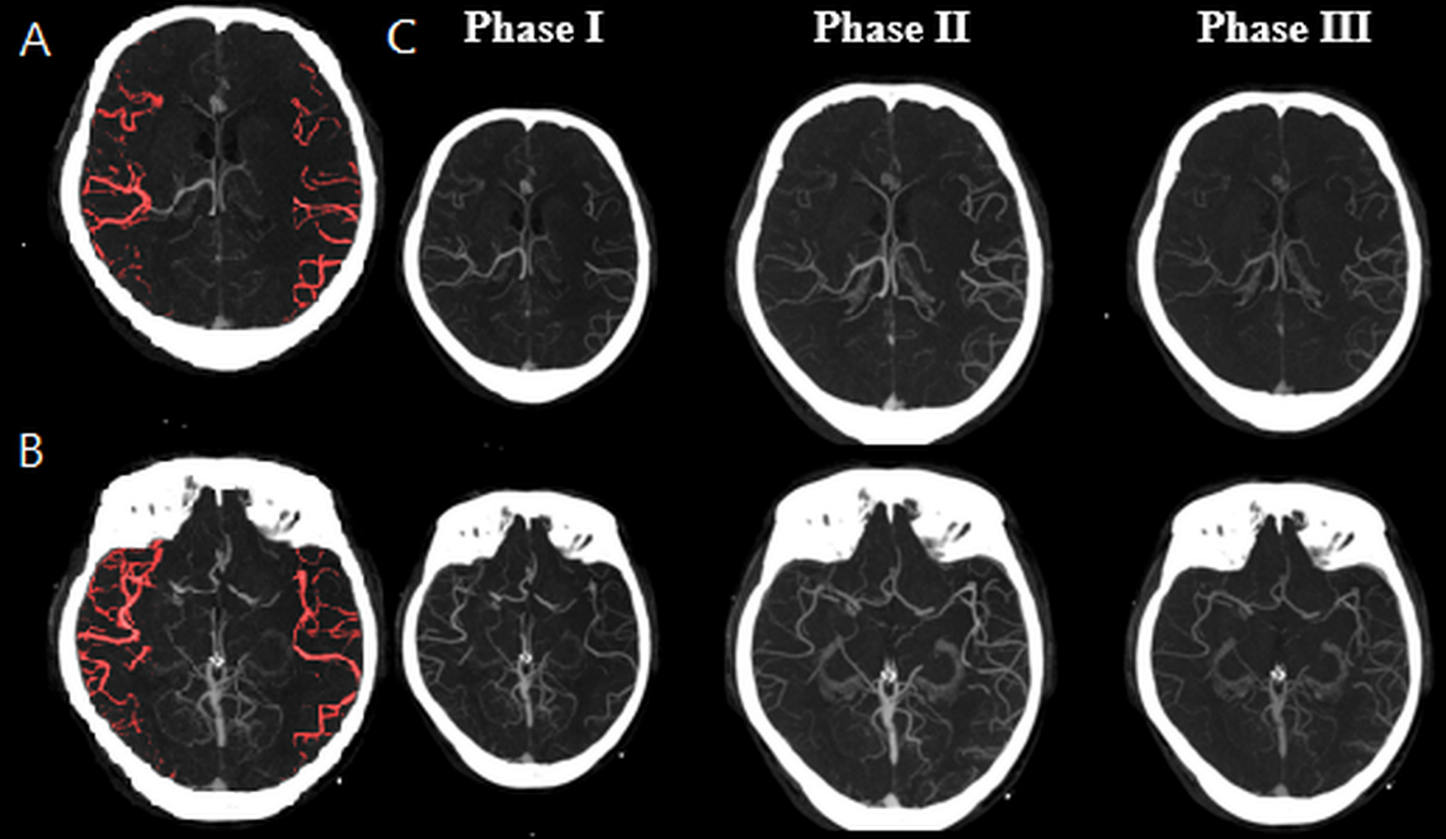
Representative axial CTA slices illustrating concordance between FD-based and visual collateral assessments. The patient, a 73-year-old male with left hemisphere occlusion and a baseline NIHSS score of 21, demonstrated consistent collateral evaluations across modalities: vCS-sCTA = 4, vCS-mCTA = 4, and mFD = 0.961. He achieved successful reperfusion (TICI 3) and a favorable functional outcome at 90 days (mRS = 1), highlighting the alignment between mFD and vCS-mCTA in identifying adequate collateral circulation. Abbreviations: vCS-sCTA, visual collateral score based on single-phase CTA; vCS-mCTA, visual collateral score based on multiphase CTA; mFD, mean fractal dimension ratio; NIHSS, National Institutes of Health Stroke Scale; TICI, Thrombolysis in Cerebral Infarction; mRS, modified Rankin Scale.

In logistic regression analyses (Table 2), an mFD > 0.9307 was associated with increased odds of favorable outcome (mRS ≤ 2). In unadjusted analysis, mFD > 0.9307 increased the likelihood of a favorable outcome sevenfold (OR = 6.93, *P* = 0.002). This association remained significant in multivariate analysis (OR = 8.13, *P* = 0.005; Table 2, Model 1), even after adjusting for age, gender, pre-NIHSS scores, and LDL levels. These findings further support mFD as a valuable prognostic indicator that may complement established criteria for EVT candidate selection. Additionally, vCS-mCTA demonstrated predictive value (adjusted OR = 3.32, *P* = 0.011; Table 2, Model 2). The mFD showed an AUC of 0.72 (*P* < 0.001), which was comparable to that of vCS-mCTA (AUC = 0.71, *P* = 0.01), suggesting similar discrimination in predicting favorable EVT outcomes. DeLong’s test indicated that this difference was not statistically significant (*P* = 0.847), supporting statistical equivalence between the two methods. Meanwhile, vCS-sCTA did not achieve statistical significance in predicting functional outcomes (*P* = 0.074), likely due to its limited temporal resolution. Figure 2 illustrates a discordant case in which vCS-sCTA underestimated collateral adequacy (score = 2), whereas both vCS-mCTA (score = 4) and mFD (0.871) indicated favorable perfusion, ultimately aligning with a good clinical outcome (90-day mRS = 0). In contrast, Fig. 3 presents a concordant case, with consistently high scores across vCS-sCTA, vCS-mCTA, and mFD, all aligned with favorable recovery. Alongside the distribution patterns observed in Fig. 1, these case-based examples highlight mFD’s potential to resolve inconsistencies inherent in static visual scoring. The mFD achieved a sensitivity of 73.9% and specificity of 71.0%, closely approximating the performance of vCS-mCTA (sensitivity: 83.9%). Although these values appear modest, they are consistent with prior collateral-based prediction studies using sCTA and reflect the intrinsic variability of collateral filling dynamics. Importantly, this level of performance supports the potential of mFD as an objective, reproducible, and clinically practical biomarker, reinforcing its feasibility as a pragmatic sCTA-based alternative—particularly in scenarios where mCTA is unavailable or inconclusive.

Despite its advantages, our FD-based collateral analysis has several limitations. First, this preliminary analysis, based on a retrospective single-center cohort with a small sample size, may be limited in statistical power and generalizability. Although relevant clinical covariates were incorporated into multivariable models to mitigate confounding, residual bias cannot be fully excluded. Accordingly, the results should be interpreted with caution. The preliminary designation in the article title reflects our recognition that the present findings represent an initial proof-of-concept requiring further validation. Future studies should focus on internal replication as well as external validation across diverse populations and imaging protocols, ideally through prospective, multicenter cohorts. Second, the mFD was derived from first-phase images acquired under an mCTA protocol using bolus tracking. While these images approximate sCTA, they are not fully equivalent to fixed-timing sCTA protocols used in routine practice. This mismatch may introduce subtle acquisition-related biases, and generalizability to real-world sCTA workflows remains to be confirmed. Third, the mFD analysis remains semi-automated and requires clinician input for selecting ganglionic and supraganglionic slices to minimize venous contamination. Although this process ensures consistent segmentation quality, future efforts should focus on full automation, PACS integration, and AI-assisted ROI selection to improve reproducibility and clinical scalability. Fourth, the optimal mFD threshold (>0.9307) was derived from the same dataset without internal or external validation, raising the possibility of overfitting. Larger, prospective multicenter studies will be necessary to confirm the generalizability of these findings. Finally, while CTP remains the reference standard for quantifying infarct core and penumbra, its availability and implementation are often limited to comprehensive stroke centers because of higher equipment cost, prolonged acquisition time, and complex post-processing. In this context, the quantitative assessment of collaterals derived from routinely available sCTA offers a pragmatic and scalable adjunct rather than a replacement for perfusion imaging. The proposed mFD approach therefore addresses an important clinical gap by providing additional, objective vascular information in hospitals without access to advanced perfusion imaging, potentially supporting early triage or transfer decisions in time-critical stroke workflows. Moreover, as the EVT time window continues to expand beyond the traditional 6–8-hour limit, the feasibility of using sCTA-equivalent FD analysis to assess collateral status in extended time window EVT patients becomes an important question for future investigation—particularly in settings where mCTA or advanced perfusion imaging is not available.

### Conclusion

This preliminary study demonstrates that the mFD, derived from sCTA-equivalent imaging, may serve as a reproducible and objective imaging biomarker for evaluating collateral status in AIS. Its predictive performance was comparable to conventional vCS-mCTA, while offering advantages in standardization, observer-independence, and usability in clinical settings where mCTA is unavailable. Although exploratory in nature, these findings suggest that mFD may assist in thrombectomy candidate selection and outcome prediction. Future studies should focus on prospective validation in larger, diverse cohorts and on the development of fully automated, AI-integrated pipelines to enable real-time implementation in acute stroke workflows.

## Supplemental Information

10.7717/peerj.21045/supp-1 Supplemental Information 1 Raw Clinical and Imaging Data for mFD-Based Collateral Assessment in AIS Patients (n=54) De-identified clinical and imaging data from 54 patients with acute ischemic stroke who underwent endovascular treatment.
Variables include the mean fractal dimension ratio (mFD), visual collateral scores from single-phase and multiphase CTA (vCS-sCTA and vCS-mCTA), age, sex, pre-treatment NIHSS scores, and 90-day modified Rankin Scale (mRS).
These data were used for logistic regression and ROC curve analysis to evaluate the prognostic value of mFD compared to conventional visual scoring methods.

## Additional Information and Declarations

### Data Availability

The following information was supplied regarding data availability: The raw data is available in the Supplemental File.
